# Supplementary material for: Observation of bosonic condensation in a hybrid monolayer MoSe2-GaAs microcavity
Source: Nat Commun. 2018 Aug 16;9:3286. doi: 10.1038/s41467-018-05532-7 (PMC6095855; doi:10.1038/s41467-018-05532-7)
Supplement: Supplementary file 1 — Supplementary Information [file 41467_2018_5532_MOESM1_ESM.pdf]

Supplementary information

Observation of bosonic condensation in a hybrid monolayer $MoSe_2$-GaAs microcavity

Max Waldherr[1]†, Nils Lundt[1]†, Martin Klaas[1]†, Simon Betzold[1], Matthias Wurdack[1], Vasilij Baumann[1], Eliezer Estrecho[2,7], Anton Nalitov[3,4,5], Evgenia Cherotchenko[4,5], Hui Cai[6], Elena A. Ostrovskaya[2,7], Alexey V. Kavokin[5,8,9], Sefaattin Tongay[6], Sebastian Klembt[1], Sven Höfling[1,7,10] and Christian Schneider[1]*

[1]Technische Physik and Wilhelm-Conrad-Röntgen-Research Center for Complex Material Systems, Universität Würzburg, D-97074 Würzburg, Am Hubland, Germany.

[2]Nonlinear Physics Centre, Research School of Physics and Engineering, Australian National University, Canberra ACT 2601, Australia

[3]Science Institute, University of Iceland, Dunhagi 3, IS-107, Reykjavik, Iceland

[4]ITMO University, St. Petersburg 197101, Russia

[5]Physics and Astronomy School, University of Southampton, Highfield, Southampton, SO171BJ, UK

[6]School for Engineering of Matter, Transport, and Energy, Arizona State University, Tempe, Arizona 85287, USA

[7]ARC Centre of Excellence in Future Low-Energy Electronics Technologies

[8]SPIN-CNR, Viale del Politecnico 1, I-00133 Rome, Italy

[9]Spin Optics Laboratory, St-Petersburg State University, 1, Ulianovskaya, 194021, Russia

[10]SUPA, School of Physics and Astronomy, University of St. Andrews, St. Andrews KY 16 9SS, United Kingdom

†These authors contributed equally to this work
**Corresponding author. Email: Christian.Schneider@physik.uni-wuerzburg.de*

Supplementary Note 1

In order to describe the eigenenergies of the hybrid polariton resonances, we apply a coupled oscillator model, which reads in the case of three oscillators:

$$\begin{bmatrix} E_{ex_1} & 0 & V_1/2 \\ 0 & E_{ex_2} & V_2/2 \\ V_1/2 & V_2/2 & E_{cav} \end{bmatrix} \begin{bmatrix} X_1 \\ X_2 \\ C \end{bmatrix} = E \begin{bmatrix} X_1 \\ X_2 \\ C \end{bmatrix}$$

the three Hopfield coefficients quantify the admixture of QW- and monolayer-exciton ($|X_1|^2$; $|X_2|^2$) and cavity photon $|C|^2$. Solving the eigenvalue problem yields the characteristic dispersion relation of hybrid polaritons, featuring three polariton branches. E_{cav} and E_{ex} are photon and exciton energies, respectively, and V_i the exciton-photon coupling strength for the respective oscillator.

Supplementary Note 2

Here, we provide details on a reference study of the power dependent behavior of the photoluminescence from pure GaAs-exciton polaritons emerging in the periphery of the monolayer device with a detuning of -5.9 meV. Sample conditions are comparable to the main text with a temperature of 5 K and optical excitation with an 82 MHz repetition rate, 2 ps pulsed Ti:Sa laser, tuned to an energy of 1.6732 eV. In Supplementary Fig. 1a-c we plot the characteristic far field spectra at injection energies of 2.4, 12 and 341 pJ/pulse. From the energy momentum dispersion relations, we can extract the energy, linewidth and the intensity input-output characteristics (see Supplementary Fig. 1), by integrating the ground state from k = -0.1 μm^{-1} to 0.1 μm^{-1} and fitting it with a Lorentz function. At an approximate average pump power of 182 pJ/pulse, we observe a distinct super linearity in the emission intensity in panel d, with a threshold-like s-shape indicative for a laser-like transition. The emission energy of the studied

mode after threshold is approximately pinned to the empty cavity resonance [see panels c and f]. This feature is typically attributed to a transition from the strong- to the weak coupling regime, which typically occurs at or slightly above the Mott density in high-quality QW-microcavities. The input power approximately corresponds to the second threshold observed in the main text, attributed to the Mott-transition of the bare GaAs QW directly in the vicinity to the monolayer.

Supplementary Note 3

This section compares the polarization of the emission from our hybrid monolayer-GaAs device with the emission from pure GaAs polaritons, recorded at a comparable laser-lower polariton detuning in relation to the hybrid polaritons of Fig. 4 (main text). The experiment was carried out under the same experimental conditions as described in the main text for Fig 4.

In Supplementary Fig. 2 we plot the resulting degree of circular polarization (DOCP) from the emission of the cavity, both subject to σ^+ and σ^- pumping. We note, that the DOCP does not show any distinct power dependency within the error margin, as expected in the linear regime by our Boltzmann model, and is significantly lower than the DOCP which we have record from our hybrid polariton condensate under significantly lower pump powers.

Supplementary Note 4

In this supplementary section, we provide additional details on the blueshift fit in Fig. 3f of the main text. The emission from the polariton condensate is subject to an energy shift, depending on the pumping power, both below and above the condensation threshold. At low pumping powers, the occupation of the ground state is negligible and the energy shift arises from the polariton interaction with the excitonic reservoir which builds up with increased excitation power. In the

conventional single reservoir model[1] the homogeneous reservoir density is fixed above the threshold due to the stimulated scattering into the ground state. The repulsive polariton-polariton interactions thus govern the further increase of the emission energy above the threshold, which occurs at a reduced rate compared to the large below-threshold blueshift due to polariton-exciton interactions. This model, despite having the advantage of simplicity, is not sufficient for the description of the hybrid polariton devices, where the emission blueshift depends nonlinearly on the pumping power above the lasing threshold.

To account for the hybrid nature of the microcavity we solve the coupled rate equations for the occupations of the polariton condensate mode and the two exciton reservoirs, neglecting the spin polarization and spontaneous scattering probability:

$$\frac{dN}{dt} = \left(W_1 n_1 + W_2 n_2 - \frac{1}{\tau}\right) N, \tag{1}$$

$$\frac{dn_i}{dt} = P_i - \left(W_i N + \frac{1}{\tau_i}\right) n_i. \tag{2}$$

Here, N is the condensate occupation, n_1 and n_2 are the densities of exciton reservoirs in the QW and the TMDC monolayer, respectively, W_1 and W_2 are the corresponding scattering rates, τ and τ_i are the condensate and reservoir lifetimes, and $P_i = Pg_i$ are the reservoir pumping rates. Equating the time derivatives to zero we find the stationary values of N and n_i.

Below the condensation threshold threshold ($N = 0$) we express the reservoir densities $n_i = Pg_i\tau_i$ from Supplementary Equation (2). Above the threshold ($N > 0$) both reservoir densities $n_i = Pg_i/(W_i N + \tau_i^{-1})$ are depleted by stimulated scattering into the condensate. The occupation of the latter reads:

$$N = \frac{N_1 + N_2}{2} + \sqrt{\left(\frac{N_1 - N_2}{2}\right)^2 + P^2\tau^2 g_1 g_2}, \tag{3}$$

where $N_i = Pg_i\tau - W_i^{-1}\tau_i^{-1}$ has the physical meaning of the occupation of a condensate, which is only coupled to the i-th reservoir. The total blueshift is governed by the polariton condensate interaction with itself and with the two reservoirs as follows:

$$\Delta E = (\alpha_1|X_1|^4 + \alpha_2|X_2|^4)N + \alpha_1|X_1|^2 n_1 + \alpha_2|X_2|^2 n_2, \quad (4)$$

where X_1 and X_2 are the exciton Hopfield coefficients of the polariton mode, corresponding to the QW and TMDC exciton, respectively.

The fits of the experimental dependence of blueshift on the pumping power, obtained with Supplementary Equations (1, 2) and (4) are shown in Fig. 3f. The initial nonlinear increase in the blueshift above the threshold corresponds to a redistribution of the excitonic densities between the two reservoirs, so that the total condensate gain provided by stimulated scattering remains equal to the radiative losses. Note that the two reservoirs model captures the nonlinear behavior of blueshift more accurately than the conventional single reservoir model[1], demonstrating the important role of the interplay of the two reservoirs in the behavior of the hybrid polariton laser. The following realistic parameters were used in the fit: $\tau = 0.5$ ps, $\tau_1 = 500$ ps, $\tau_2 = 10$ ps. We also assume equal excitonic interaction constants[2] $\alpha_1 = \alpha_2$ and take equal Hopfield coefficients $X_1 = X_2$ from the polariton branch fit in Fig. 2 of the main text. Note that, under this assumptions, the reservoir contribution to the condensate blueshift is proportional to the total reservoir density $n = n_1 + n_2$. The free parameters used for the fit are the two relations between the pumping efficiencies $g_1/g_2 = 1$ and the scattering rates $W_1/W_2 = 0.02$.

The details of the fit presented in Fig. 3f are shown in Supplementary Fig. 3. Below the threshold, the blueshift is linear in the pumping power. The population of QW excitons builds faster than in the TMDC due to longer exciton lifetime in the QW. Above the threshold, both reservoirs are depleted by stimulated scattering into the macroscopically populated polariton mode. Depletion efficiency is governed by the spontaneous scattering rate. Stronger coupling of the TMDC

excitonic mode to the polaritonic mode favors stronger TMDC reservoir depletion and results in a slight decrease of its occupation above the threshold. In contrast, the QW reservoir grows so that the total gain provided by the two reservoirs compensates the radiative losses of the condensate. This results in a nonlinear dependence of the blueshift on the pumping power above the threshold. At higher powers, however, the combined reservoir density reaches a steady state, and the blueshift becomes linear because it is governed by polariton-polariton interactions as in the single-reservoir case[1].

It is straight forward to quantify the threshold condition of the hybrid polariton laser, applying Supplementary equations 1 and 2. Assuming, as bove, that both reservoirs are pumped with the same efficiency $\frac{g_1}{g_2} = 1$, the threshold reservoir injection rate for the hybrid condensation obeys the condition:

$$\frac{1}{P_{th}} = \tau \left[W_1\tau_1 + W_2\tau_2\right] = \frac{1}{P_{QW}} + \frac{1}{P_{TMDC}}, \tag{5}$$

Where P_{QW} and P_{TMDC} are condensation thresholds for the QW and TMDC polaritons, respectively. This means that the pump threshold in the hybrid structure is always lower than that in a QW or TMDC layer alone. Physically this is due to the fact that both reservoirs scatter into the same polariton state, so the reservoir densities required to overcome polariton decay are much lower.

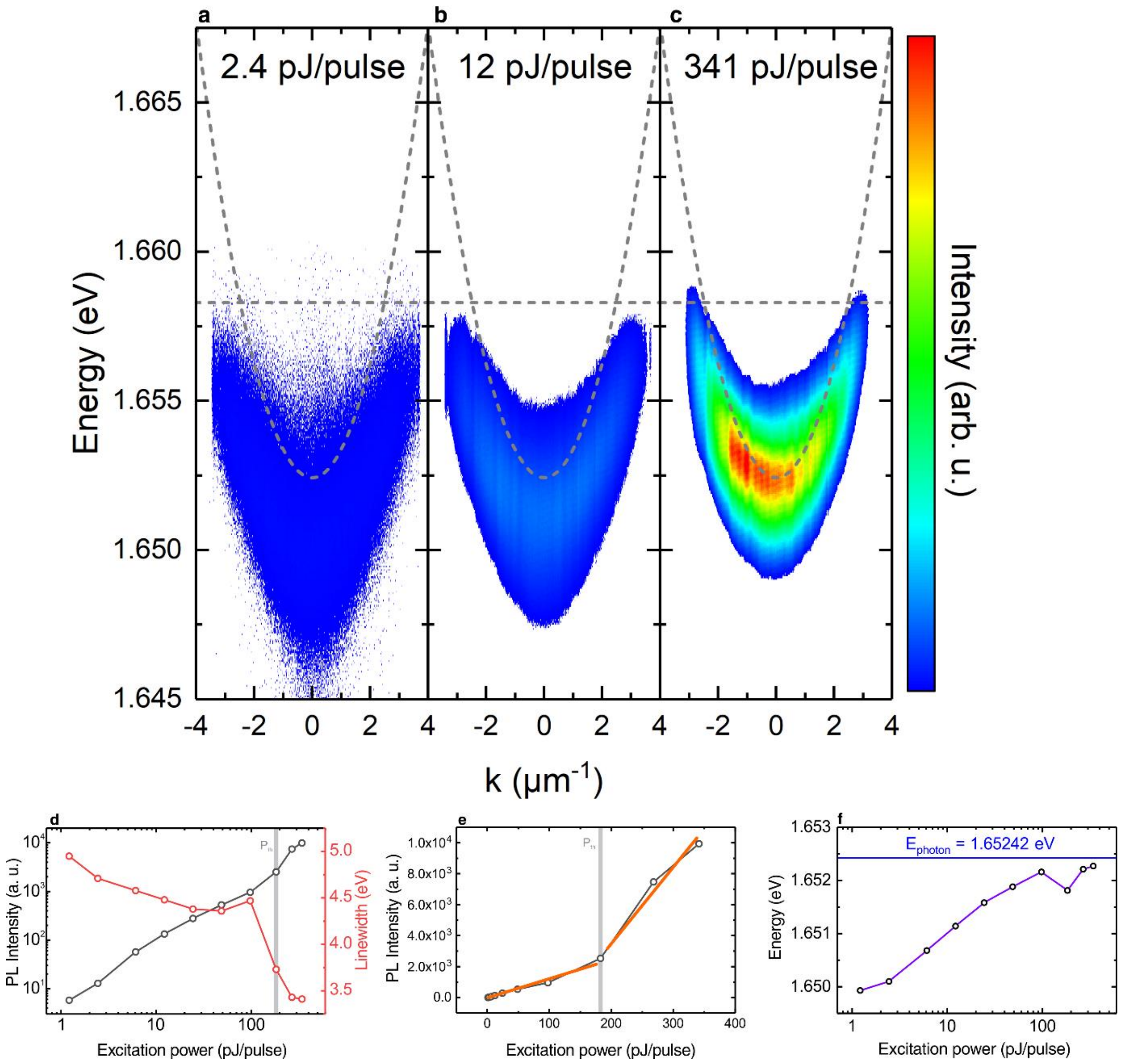

Supplementary Figure 1 | False color intensity profile of the pure GaAs device at a detuning of –5.9 meV at different excitation powers. **a,b,** Lower polariton emission under the threshold (2.4 and 12 pJ/pulse, respectively) and **c,** photon lasing above the threshold (341 pJ/pulse). The flat dashed line (parabolic dashed line) corresponds to the exciton mode (photon mode). **d,** PL intensity (black) with the distinct s-shape and linewidth (red) as a function of excitation power. In contrast to the hybrid device, we observe only one threshold at much higher pump powers, corresponding to the conventional inversion based lasing transition. **e,** PL intensity as a function

of excitation power on a linear scale. **f,** PL emission energy (blue) as a function of the excitation power.

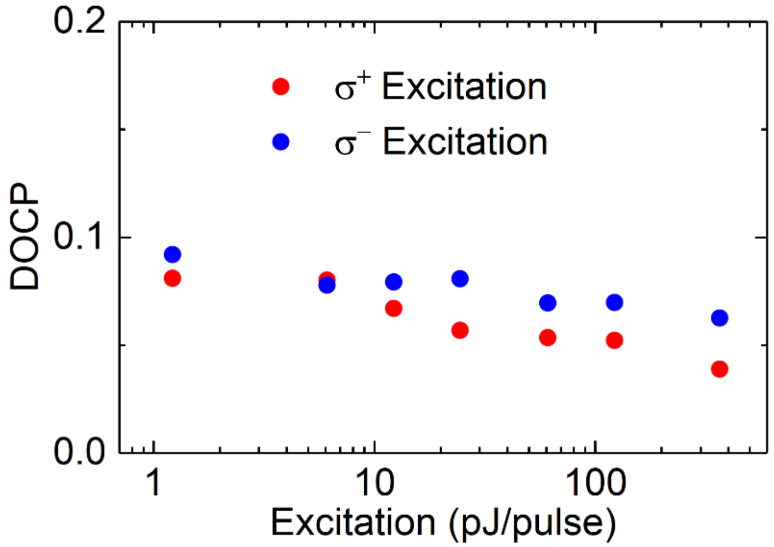

Supplementary Figure 2 | Degree of circular polarization from the pure GaAs lower polariton branch for both σ^+ (red) and σ^- (black) polarized excitation for comparable laser-LP detuning (in relation to the hybrid GaAs-$MoSe_2$ LP) depending on excitation power. We observe no increase even for strong laser pump and the DOCP is significantly lower than in the condensate phase of the hybrid GaAs-$MoSe_2$ device displayed in the main text, which substantiates the claim regarding polarization conservation due to bosonic condensation in a hybrid $MoSe_2$-GaAs microcavity.

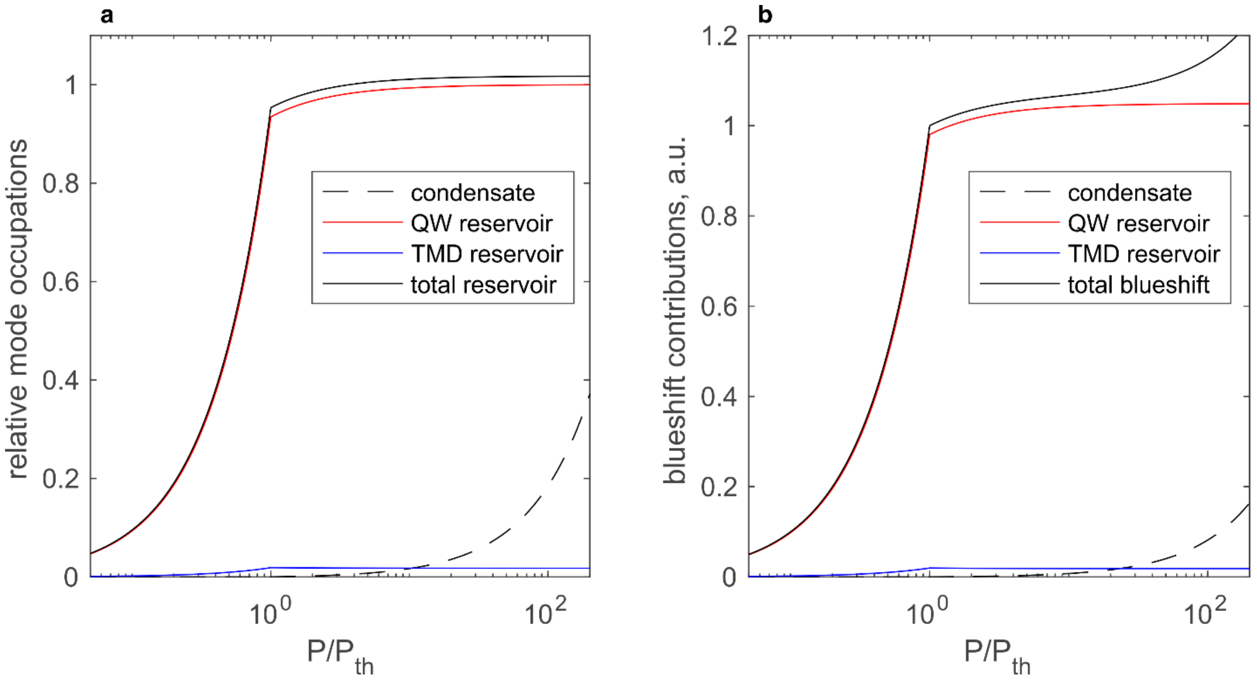

Supplementary Figure 3 | a, Relative polariton condensate and reservoir occupations. **b,** Relative contributions to the total emission blueshift from the condensate self-interaction and the two reservoirs.

References:

1. Su, R. *et al.* Room-Temperature Polariton Lasing in All-Inorganic Perovskite Nanoplatelets. *Nano Lett.* **17,** 3982–3988 (2017).

2. Shahnazaryan, V., Iorsh, I., Shelykh, I. A. & Kyriienko, O. Exciton-exciton interaction in transition-metal dichalcogenide monolayers. *Phys. Rev. B* **96,** 115409 (2017).
